# A biomechanical analysis of anterior cervical discectomy and fusion alone or combined cervical fixations in treating compression-extension injury with unilateral facet joint fracture: a finite element study

**DOI:** 10.1186/s12891-021-04814-4

**Published:** 2021-11-10

**Authors:** Chen Jin, Zhong Wang, Peng Liu, Yaoyao Liu, Zhanwei Wang, Ning Xie

**Affiliations:** 1 Division of Spine Surgery, Department of Orthopedics, Tongji Hospital, Tongji University School of Medicine, 389 Xincun Road, Putuo District, Shanghai, 200065 China; 2 Division of Spine Surgery, Department of Orthopedics, Daping Hospital of Army Medical University, Chongqing, China; 3 State Key Laboratory of Trauma: Burns & Combined Wound, Institute for Traffic Medicine of Army Medical University, Chongqing, China

**Keywords:** Compression-extension injury, Facet joint fracture, Anterior cervical discectomy and fusion, Combined cervical fixations, Spine surgery, Biomechanics, Finite element analysis

## Abstract

**Background:**

Compression-extension injury with unilateral facet joint fracture is one of the most devastating injuries of subaxial cervical spine. However, it is not yet clear which fixation technique represents the optimal choice in surgical management. This study aims to assess the construct stability at the operative level (C4/C5 cervical spine) following anterior cervical discectomy and fusion (ACDF) alone and combined fixation techniques (posterior-anterior fixations).

**Methods:**

A previously validated three-dimensional C2-T1 finite element model were modified to simulate surgical procedures via the anterior-only approach (ACDF) and combined cervical approach [(transarticular screw, lateral mass screw, unilateral pedicle screw, bilateral pedicle screw) + ACDF, respectively] for treating compression-extension injury with unilateral facet joint fracture at C4/C5 level. Construct stability (range of rotation, axial compression displacement and anterior shear displacement) at the operative level was comparatively analyzed.

**Results:**

In comparison with combined fixation techniques, a wider range of motion and a higher maximum von Mises stress was found in single ACDF. There was no obvious difference in range of motion among transarticular screw and other posterior fixations in the presence of anterior fixation. In addition, the screws inserted by transarticular screw technique had high stress concentration at the middle part of the screw but much less than 500 MPa under different conditions. Furthermore, the variability of von Mises stress in the transarticular screw fixation device was significantly lower than ACDF but no obvious difference compared with other posterior fixations.

**Conclusions:**

Of the five fixation techniques, ACDF has proven poor stability and high structural stress. Compared with lateral and pedicle screw, transarticular screw technique was not worse biomechanically and less technically demanding to acquire in clinical practice. Therefore, our study suggested that combined fixation technique (transarticular screw + ACDF) would be a reasonable treatment option to acquire an immediate stabilization in the management of compression-extension injury with unilateral facet joint fracture. However, clinical aspects must also be regarded when choosing a reconstruction method for a specific patient.

## Background

Traumatic facet joint fracture and dislocation is one of the most common devastating injuries of subaxial cervical spine (SCS) injuries [1]. The facet joint is an important load-bearing structure and the medial wall of the intervertebral foramen. Fractures of the facet joints in cervical fracture dislocations have a significant influence on spinal stability. In the previous study, it is agreed that fractures of the lateral mass and articular process were generally accepted as being produced by compression-extension injury (CEI) or hyperextension combined with a rotational injury mechanism according to Allen’s classification [2–4].

Y. Kotani et al. [5] have introduced a new classification to clarify the injury pattern as well as the degree of discoligamentous injuries in cervical lateral mass and facet joint fractures. They described the comminution-type fracture was the most severe subtype that consisted of multiple fracture lines in the lateral mass with significant fragmentations, frequently accompanied by significantly higher rates of coronal malalignment. In addition, 24% of anterior translation of fractured vertebra was observed and signal changes in intervertebral disc were demonstrated in 76% of caudal segments and 24% of cephalad segments adjacent to fractured vertebra of lateral mass fractures. In this case, they proposed that single-level posterior fixation procedure has proven poor fracture reduction and failure in repairing injured disc.

In the literature, clinical management of unstable CEI (UCEI), CEI involving unilateral facet joint fracture (UFJF) and anterior injured disc, was well accepted for the needs of cervical decompression, repair of injured disc and restoration of spinal stability. And combined anterior-posterior or posterior-anterior procedures were widely performed by the majority of surgeons. Posterior fixation techniques included pedicle screw (PS), lateral mass screw (LS) and transarticular screw (TS) techniques [6–8]. Meanwhile, a minority of surgeons were inclined to perform single anterior cervical discectomy and fusion (ACDF) [9–11]. Although the literatures supported that both two stabilizations had similar clinical outcome with good surgical results, there were advantages and disadvantages of one over the other in the management of UCEI.

To date, it is not yet clear which technique represents the optimal choice and whether stabilization devices can be efficient and beneficial for UCEI. There are two following research issues the authors mostly concern about: (1) Is it stable enough biomechanically of ACDF only? (2) Is it possible to select a more minimally invasive posterior fixation technique if performing combined cervical approach? Although previous reports have shown several finite element (FE) models of cervical spine, information about detailed structural response to external loading, especially to evaluate combined internal fixations, is still lacking [12, 13].

Therefore, the aim of this study is targeted to construct an FE model of UCEI, and to further assess the construct stability at the operative level (C4/C5 cervical spine) following ACDF only and combined cervical fixation techniques [posterior fixations (TS, LS, unilateral PS and bilateral PS) + ACDF].

## Methods

### FE modelling and validation

A previously developed and validated three-dimensional FE model of the C2-T1 ligamentous SCS was used in the current study [14]. The C4/C5 segment from this model was extracted and analyzed. The reason for choosing the C4/C5 motion segment involved that the large amount of facet injuries commonly occurred on this joint in clinical practice, its use in previous cervical spine models, and its similarity to other segments in the full spine model [15, 16]. Our previous study have showed validation of the C4/C5 FE model in detail by comparing with the experimental data obtained from normal cadaver and published data reported in the literatures [12, 17–19].

The images were segmented manually by several spine surgeons and an experienced anatomist using commercial software Adobe Photoshop (Adobe Systems, California, USA). Quality of segmentation and surface smoothing were checked using commercial software Amira (5.3.3, Visage Imaging, Carlsbad, CA). The acquired surface files were processed in HyperMesh (V12.0, Altair, Michigan, America) for meshing and refinement. The numerical model and simulations were conducted using FE software Abaqus (Simulia, Providence, RI).

**Fig. 1:**
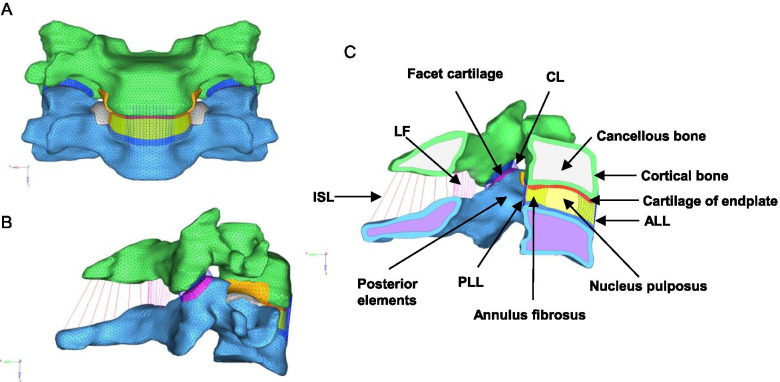
A three-dimensional finite element model of C4/C5 cervical spine and other structures and details. **a** Norma anterior, **b** Lateral view, **c** Details of the anatomical structures in sagittal plane. *ALL* anterior longitudinal ligament, *PLL* posterior longitudinal ligament, *CL* capsular ligament, *LF* ligamenta flava, *ISL* interspinous ligaments

The current FE model shown in Fig. 1 consisted of cancellous bone, cortical bone, cartilage of endplate, annulus fibrosus, nucleus pulposus, facet cartilage, posterior elements, anterior longitudinal ligament (ALL), posterior longitudinal ligament (PLL), capsular ligament (CL), ligamenta flava (LF) and interspinous ligaments (ISL).

Vertebral bodies and posterior elements were modeled using solid elements, but the material was described as isotropic. Cortical and cancellous bone was taken into consideration, and modeled using isotropic elastic four-node tetrahedral elements. Facet cartilage was modeled using hexahedral elements with an isotropic-elastic material model. The facet joint was modeled with a surface contact algorithm and friction coefficient was assumed to be 0.1 [20].

The intervertebral disc was modeled as a central nucleus with a surrounding ring-shaped annulus, covered with two cartilaginous endplates. Cartilaginous endplate was modeled with eight-node isotropic-elastic solid elements. The nucleus was modeled using hyper-elastic, incompressible, two-parameter Mooney-Rivlin formulation [21].

Five major ligaments approximating the ligamentous structures of cervical spine based on human anthropometry were incorporated into the FE model as two-node, tension-only and nonlinear spring elements [22, 23].

A total of 62,546 nodes and 187,650 elements were used to build the C4/C5 segment model so as to incorporate the full details of the complicated cervical geometries. The material properties for the model structures and instrumentations (titanium) were taken from the literature as shown in Table 1 [13, 21, 24–27].

**Table 1:** Material properties of the spinal structures and instrumentations

Component	Material model	Element type	Material property	Reference
Cortical bone	ISO elastic	C3D4	E = 12,000 Mpa, μ = 0.3	[21]
Cancellous bone	ISO elastic	C3D4	E = 300 Mpa, μ = 0.3	[21]
Posterior elements	ISO elastic	3-D solid	E = 3500 Mpa, μ = 0.3	[13]
Cartilaginous endplate	ISO elastic	C3D8	E = 23.8 Mpa, μ = 0.3	[19]
Cartilage of joint	ISO elastic	C3D8	E = 23.8 Mpa, μ = 0.3	[19]
Nucleus pulposus	Hyper-elastic	C3D8H	C10 = 0.12, C01 = 0.09	[19]
Annulus ground substance	Hyper-elastic	C3D8H	C10 = − 0.075 Mpa	[23]
			C01 = 0.122 Mpa	
			C20 = − 0.294 Mpa	
			C11 = 0.689 Mpa	
			C02 = 0.122 Mpa	
Annulus fibrosus	NON-linear Spring	SpringA	Stress-strain curve	[19, 22, 24]
Ligaments	NON-linear Spring	SpringA	Force-deflection curve	[20, 25]
Bone graft	ISO elastic	3-D solid	E = 3500 Mpa, μ = 0.3	[13]
Instrumentations (titanium)	ISO elastic	3-D solid	E = 110,000 Mpa, μ = 0.3	[13]

*E* Young’s modulus, *μ* Poisson's ratio, *C*_*ij*_*, D* material constant, *ISO* isotropic

### Surgery simulation

**Fig. 2:**
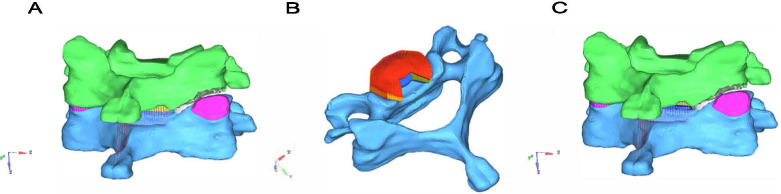
The surgery-simulated finite element model. **a** Right cervical articular joint of C5 was removed. **b** Posterior half of the disc and posterior longitudinal ligament were removed. **c** Compression-extension injury with unilateral facet joint fracture finite element model (**a** + **b**)

All models were based on a validated model of the aforementioned intact C4/C5 level. It was then imported into the FE package Abaqus to build the UCEI simulation model. Right cervical articular joint of C5, the posterior half of the disc and PLL were removed (Fig. 2). We deleted the corresponding structures to simulate UCEI more precisely.

**Fig. 3:**
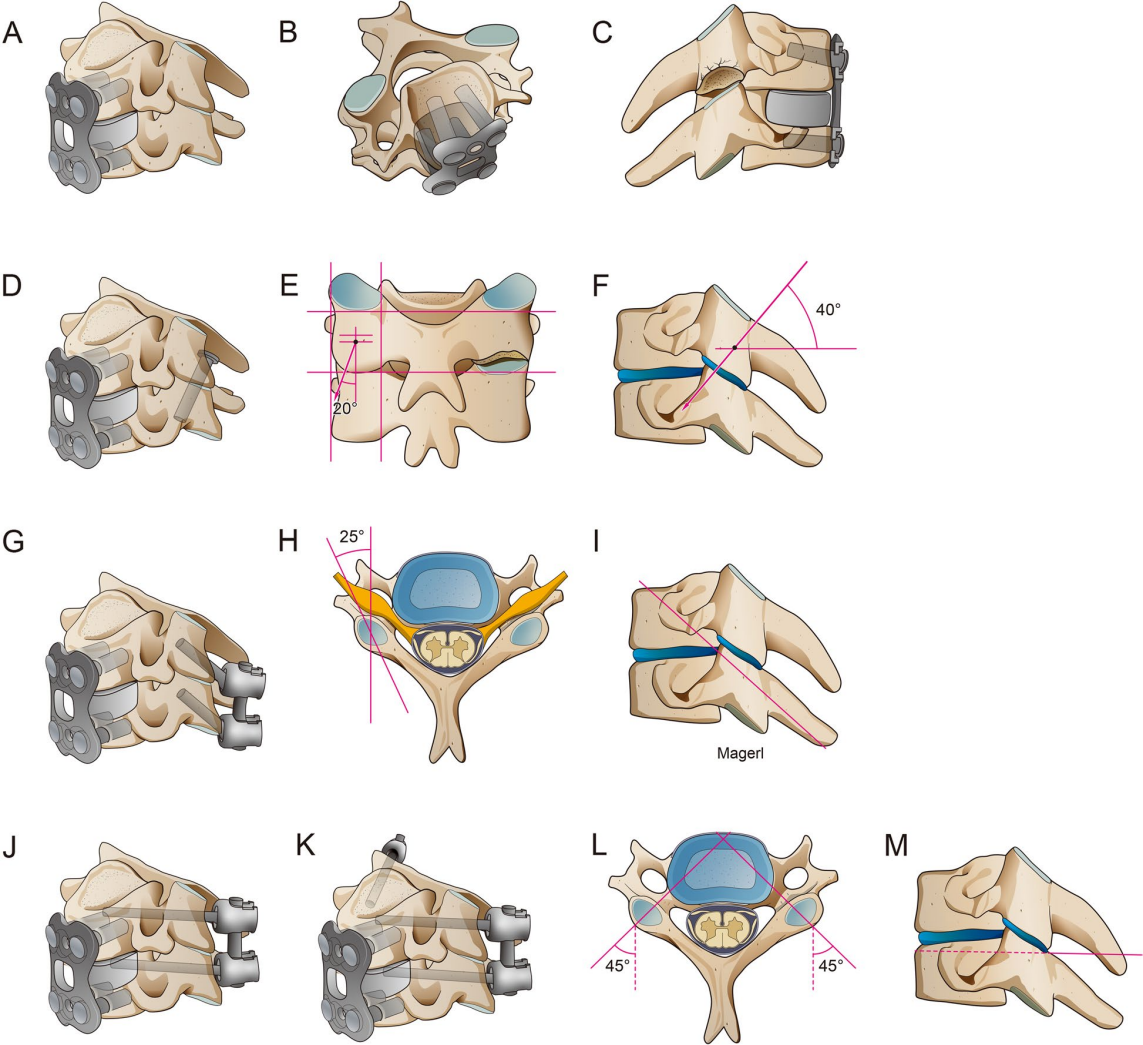
The presentation of internal fixation system and techniques. **a-c** Anterior plate-screw fixation technique, top-down view and right lateral view. **d-f** Comined anterior plate-screw fixation and posterior transarticular screw technique, and inward and outward direction of screw insertion. **g-i** Comined anterior plate-screw fixation and posterior lateral mass screw technique, and inward and outward direction of screw insertion. **j-m** Comined anterior plate-screw fixation and unilateral or bilateral pedicle screw technique, and inward and outward direction of screw insertion

A total of five surgery-simulated FE models of UCEI, including four models simulating posterior-anterior cervical fixations and one model simulating ACDF alone, were built. The internal fixation systems were implanted with anterior plate-screw fixation system and four posterior fixation techniques (TS, LS, unilateral PS and bilateral PS) in the models (Fig. 3). To simulate ACDF at C4/C5 level, the ALL, intervertebral disc and cartilaginous endplate were removed. What’s more, a cylindrical strut bone graft was placed between the intervertebral space occupying 50% of the opposing endplate areas [13]. The bone grafts were not rigidly fused to the relative endplate and allowed compression but not tension transmission. After bone graft placement, a titanium plate (height 21-24 mm, width 10 mm, and thickness 2 mm) was rigidly fixed to screws from C4 to C5 to provide additional stability to the fixation model. Along the ends of the anterior plate, two titanium screws were placed inside both C4 and C5 vertebral bodies within 1.00-mm distance from the end plates. Screws of 14-mm length with a mean diameter of 3.5 mm were rigidly fixed in the operative segment (Fig. 3A, B, C). The plate-screw and screw-bone interface were fully constrained for all six degrees of freedom. The screws were inserted parallel to the superior endplate and the medial inclination angle to the sagittal plane was approximately 13°. Surface to surface contact algorithm between the bone graft and adjacent endplate was used to describe interaction and the friction coefficient was set as 0.4. Screws were assumed to have the same dimensions, drilling depth, drilling angle.

To simulate posterior fixation system, we confirmed the size and location of screws and rods to obtain the appropriate internal fixation systems (Fig. 3): (1) TS technique: The entry point was 1 mm medial to the center of the lateral mass in the C4, with the screw at a downward direction of 40° in the sagittal plane and at an outward direction of 20° in the coronal plane [28] (Fig. 3D, E, F). (2) LS technique: The entry point was 3 mm medial and superior to the center of the lateral mass, with the screw at an outward direction of 25° from the vertical line and upward direction parallel to the superior facet joint [29] (Fig. 3G, H, I). (3) PS technique: The entry point was 2 mm below the base of the superior articular process, with the screw at an inward direction of 45° in the horizontal plane [30] (Fig. 3J, K, L, M). The internal fixation system material was set as titanium and modelled as linear elastic isotropic. The contact surfaces of the screws and screw holes were simulated by making rough enough (infinite friction coefficient) in order to prevent extraction. The bone fusion was not taken into consideration since four posterior fixation models were simulated the postoperative stage immediately.

### Biomechanical comparison

The C4/C5 construct stability was measured in terms of range of motion (ROM), the compression displacement and the anterior shear displacement: (1) ROM: a 2.0-Nm quasi-static pure moment (flexion, extension, lateral bending and axial rotation) was applied to the superior surface of C4. (2) The axial compression displacement: a compressive force of 50.0 N was applied in the vertical downward direction at the superior endplate of the C4 vertebral body. (3) The anterior shear displacement: a 50.0 N of anterior shear loading was applied at the superior endplate of the C4 vertebral body. The inferior surface of C5 was fully constrained in all conditions.

Stress analyses were carried out and the stress distribution of von Mises were compared among various fixation devices to predict the tendency of fracture according to the fixation techniques.

## Results

### Kinematics analyses

**Fig. 4:**
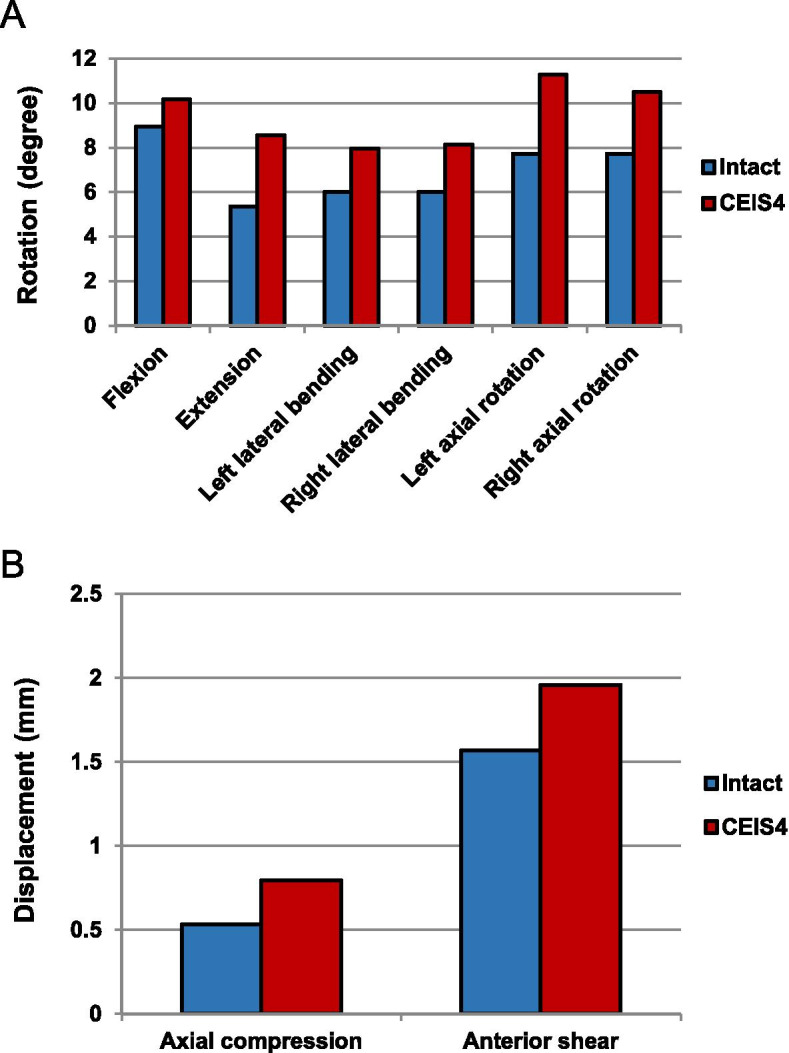
The motions of intact and compression-extension injured models under flexion-extension, lateral bending, axial rotation, axial compression and anterior shear modes. **a** Range of rotation, **b** Displacement of axial compression and anterior shear

The kinematics analyses of the intact and UCEI models in C4/C5 local segment were shown in Fig. 4. The ROM between the intact model and UCEI model were: flexion (8.94°, 10.17°), extension (5.38°, 8.56°), left lateral bending (6.01°, 7.94°), right lateral bending (6.01°, 8.13°), left axial rotation (7.73°, 11.27°), and right axial rotation (7.73°, 10.49°), respectively (Fig. 4A). The values of displacement were axial compression displacement (0.53 mm, 0.79 mm) and anterior shear displacement (1.57 mm, 1.96 mm) (Fig. 4B). Compared with the intact model, the UCEI model exhibited higher motion as well as displacement: flexion (13.8%), extension (59.1%), left lateral bending (32.1%), right lateral bending (35.3%), left axial rotation (45.8%), right axial rotation (35.7%), axial compression displacement (49.1%), and anterior shear displacement (19.9%), respectively.

**Fig. 5:**
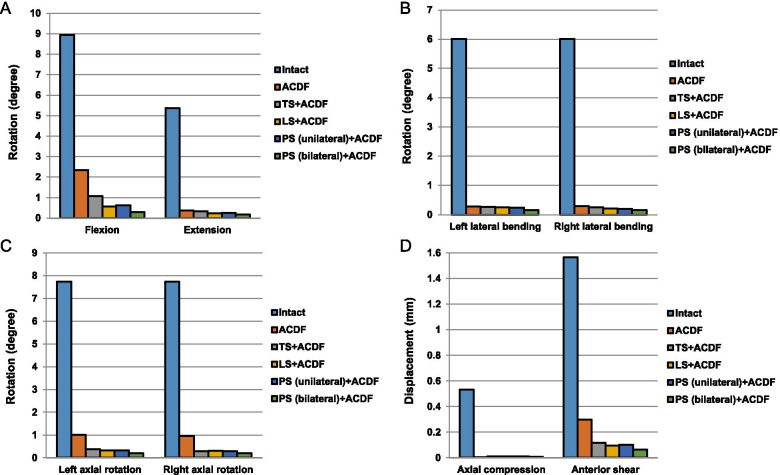
The motions of surgical-simulated models under flexion-extension, lateral bending, axial rotation, axial compression and anterior shear modes. **a-c** Range of rotation, **d** Displacement of axial compression and anterior shear

The kinematics analyses of the intact model and five surgery-simulated FE models were shown in Fig. 5. Flexion, extension, lateral bending (left/right), and axial rotation (left/right) motions from C4 to C5 for the FE models [intact, ACDF, TS + ACDF, LS + ACDF, PS (unilateral) + ACDF, PS (bilateral) + ACDF] were recorded to be (8.94°, 2.36°, 1.07°, 0.57°, 0.63°, 0.30°), (5.38°, 0.37°, 0.33°, 0.24°, 0.26°, 0.18°), (6.01°/6.01°, 0.29°/0.29°, 0.27°/0.25°, 0.25°/0.21°, 0.24°/0.20°, 0.16°/0.16°), (7.73°/7.73°, 1.01°/0.95°, 0.38°/0.28°, 0.32°/0.30°, 0.31°/0.29°, 0.20°/0.19°), respectively (Fig. 5A, B, C). As for axial compression displacement and anterior shear displacement, the values were recorded to be (0.53 mm, 0.00 mm, 0.01 mm, 0.01 mm, 0.01 mm, 0.01 mm) and (1.57 mm, 0.30 mm, 0.12 mm, 0.10 mm, 0.10 mm, 0.06 mm) (Fig. 5D). Compared with the intact model, the ROM and displacement in other five surgical models were lower in each condition: flexion (73.6, 88.0, 93.6, 93.0, 96.6%), extension (93.1, 93.9, 95.5, 95.2, 96.7%), left lateral bending (95.2, 95.5, 95.8, 96.0, 97.3%), right lateral bending (95.2, 95.8, 96.5, 96.7, 973%), left axial rotation (86.9, 95.1, 95.9, 96.0, 97.4%), right axial rotation (87.7%, 96.4, 96.1, 96.2, 97.5%), axial compression displacement (100, 98.1, 98.1, 98.1, 98.1%), and anterior shear displacement (80.9, 92.4, 93.6, 93.6, 96.2%). In summary, the C4/C5 segment lost more than 85% of its motion after combined plate-screw fixations in flexion and more than 90% in other conditions.

### Stress analyses

Qualitative investigation of the stress characteristics on fixation devices can predict the tendency of fracture according to the fixation techniques. The effect of fixation location on load transfer can also be evaluated from the quantified result of stress concentration.

**Fig. 6:**
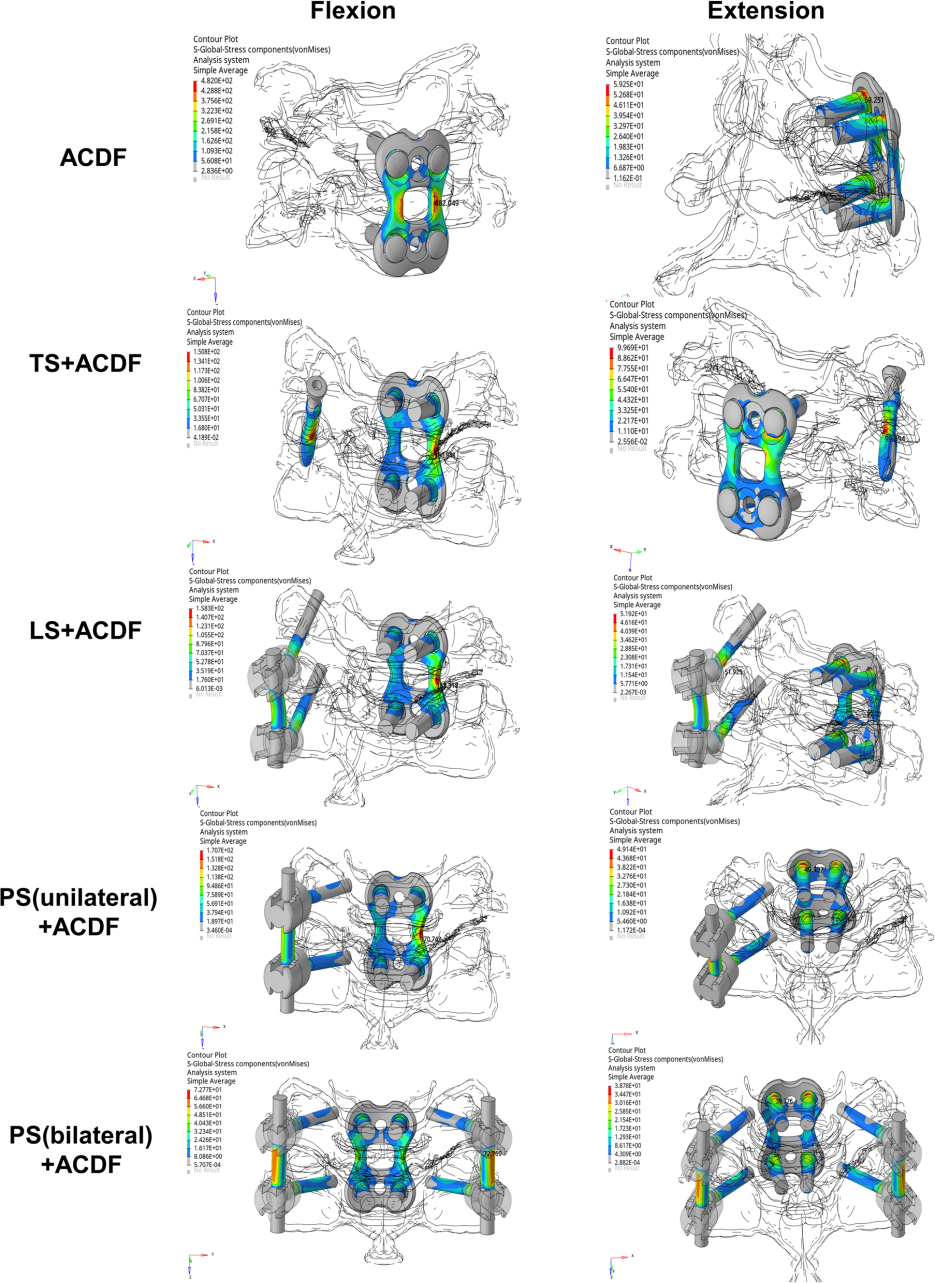
The von Mises stress distribution of the fixation devices under flexion and extension conditions

**Fig. 7:**
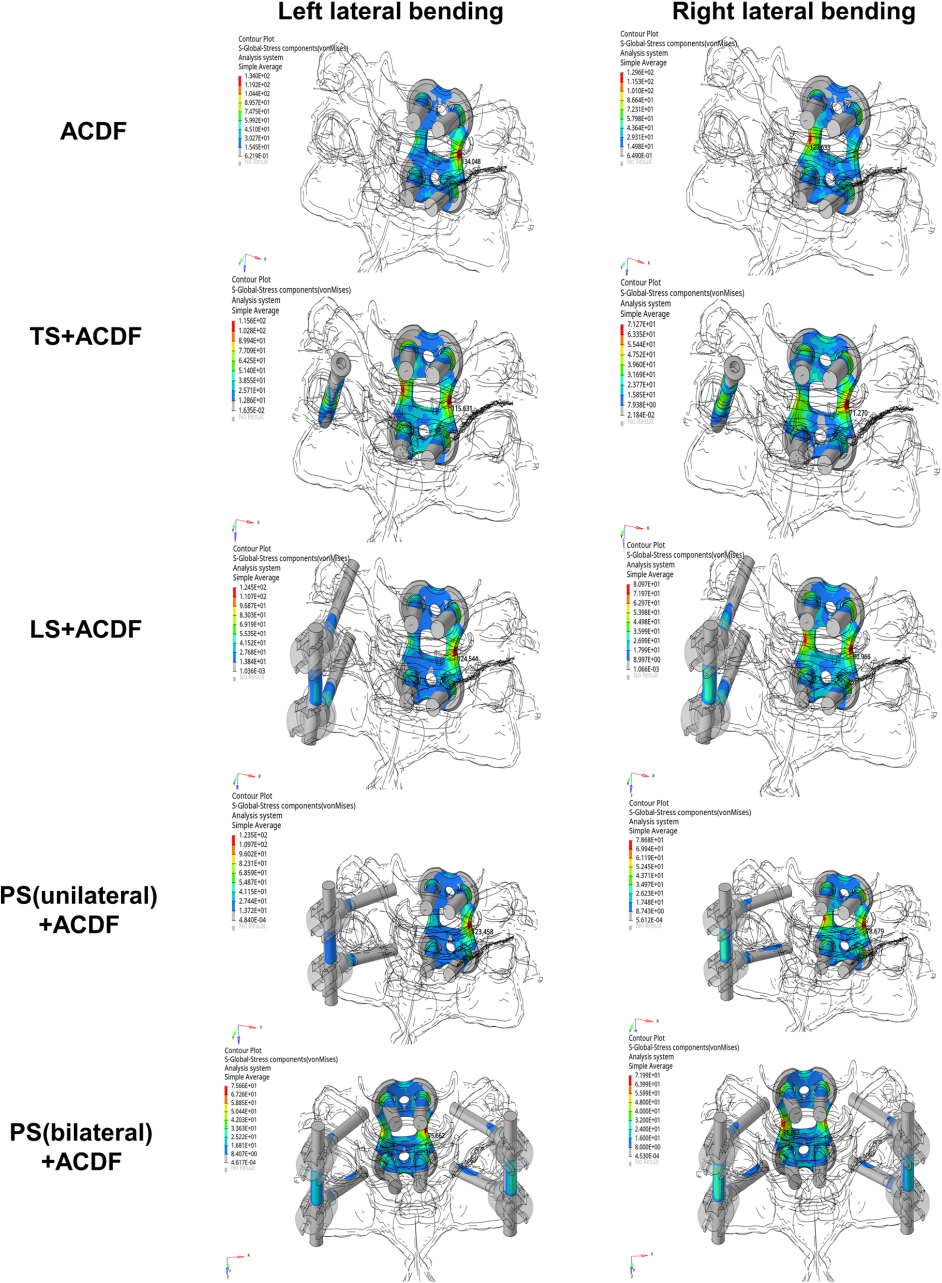
The von Mises stress distribution of the fixation devices under lateral condition

**Fig. 8:**
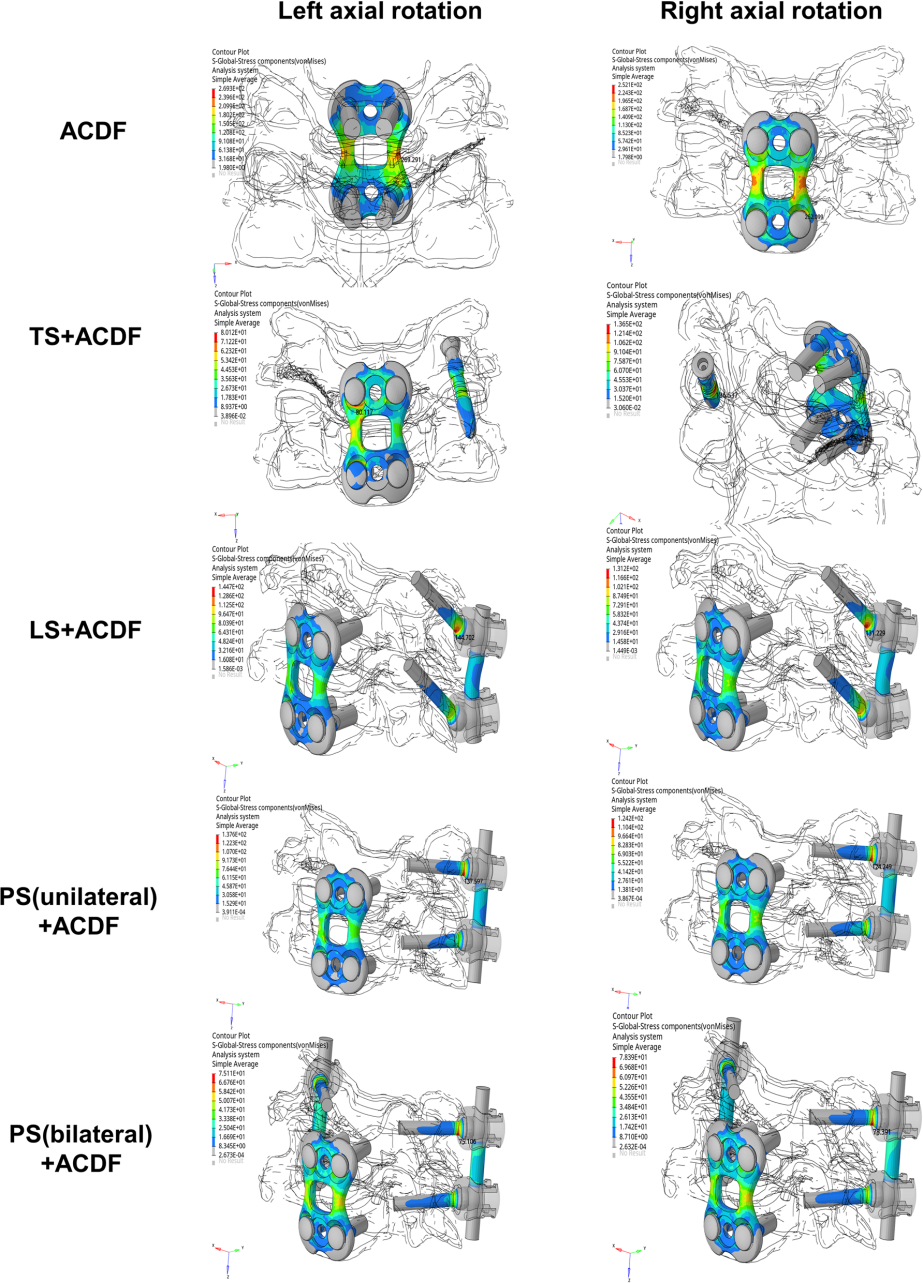
The von Mises stress distribution of the fixation devices under axial rotation condition

Under different conditions, the maximum von Mises stress of instrumentation differed based on different surgery-simulated models (Figs. 6, 7 and 8). (1) Under flexion, high stress concentrations were observed at the middle part of anterior plate when performed ACDF (482.05 MPa), TS + ACDF (251.47 MPa), LS + ACDF (158.32 MPa) and PS (unilateral) + ACDF (170.74 MPa); and at the cap-rod-screw interface when performed PS (bilateral) + ACDF (72.38 MPa). (2) Under extension, high stress concentrations were observed at the upper bone-screw interface of anterior plate-screw fixation system when performed ACDF (59.25 MPa), TS + ACDF (62.47 MPa), PS (unilateral) + ACDF (49.14 MPa) and PS (bilateral) + ACDF (38.78 MPa); and at the upper cap-rod-screw interface when performed LS + ACDF (51.93 MPa). (3) Under lateral bending (left/right), the maximum stress was found at the middle part of anterior plate in all models: [ACDF (134.05 MPa /129.63 MPa), TS + ACDF (118.66 MPa /97.27 MPa), LS + ACDF (124.54 MPa /80.97 MPa) and PS (unilateral) + ACDF (123.46 MPa /78.68 MPa), PS (bilateral) + ACDF (75.66 MPa /71.99 MPa)]. (4) Under left axial rotation, the maximum stress was found at the middle part of anterior plate in ACDF model (269.29 MPa); at the upper bone-screw interface of anterior plate-screw system in TS + ACDF (138.18 MPa); and at the upper cap-rod-screw interface in LS + ACDF (144.70 MPa) and PS (unilateral) + ACDF (137.60 MPa), PS (bilateral) + ACDF (75.11 MPa). (5) Under right axial rotation, the maximum stress was found at the middle part of anterior plate in ACDF model (252.10 MPa); at the posterior bone-screw interface in TS + ACDF (85.45 MPa); and at the upper cap-rod-screw interface in LS + ACDF (131.23 MPa) and PS (unilateral) + ACDF (124.25 MPa), PS (bilateral) + ACDF (78.39 MPa). The single use of anterior plate-screw fixation demonstrated maximal stress level was nearly 500 MPa in flexion, obviously higher than combined fixation techniques. And stress concentration on anterior construct decreased following various posterior constructs performed, which avoided the risk of screw or plate breakage.

**Fig. 9:**
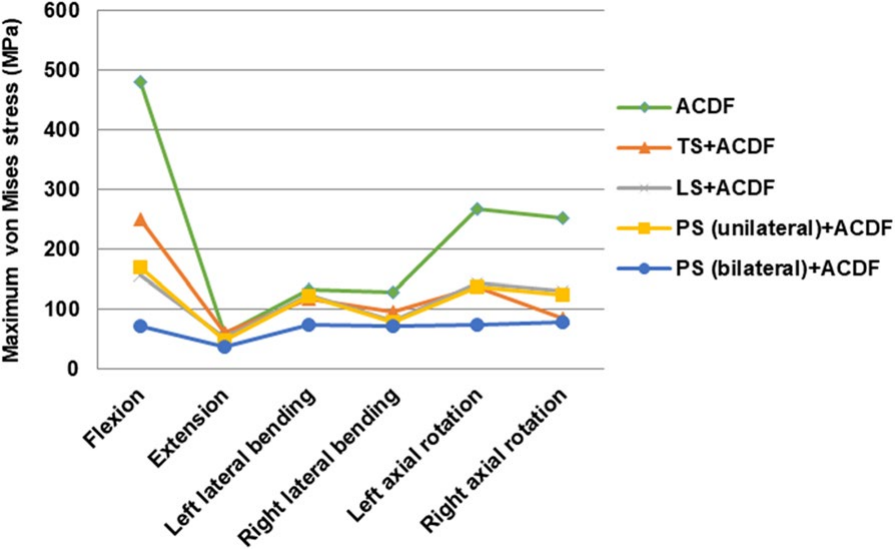
Comparison of maximum von Mises stresses on fixation devices in flexion-extension, lateral bending, axial rotation modes

Figure 9 showed maximal von Mises stress comparisons among the fixation techniques in flexion, extension, left-right lateral bending, and left-right axial rotation conditions. Under flexion-extension conditions, the maximum von Mises stress results showed that the variability of stress in the ACDF device was maximum and PS (bilateral) fixation device was minimum. However, there were no obvious differences among TS, LS and PS (unilateral) techniques. Under lateral bending and axial rotation conditions, the implants showed no significant differences in the variability of von Mises stress.

## Discussion

Injuries to the SCS account for the majority of cervical injuries, making up about 65% of fractures and more than 75% of all dislocations [31]. Classification of SCS is difficult because of the complex anatomy of vertebrae, the presence of a three-joint complex, and many ligamentous structures responsible for stability. Vaccaro et al. [32] reported that disruption of the herniated nucleus pulposus was associated with 56% of unilateral facet dislocations and 82% of bilateral facet dislocations. To date, the debate on optimal reduction and fixation of SCS with unilateral facet dislocation or fracture via surgical procedures still remains unclear. Although unilateral facet dislocation in distractive flexion injuries (DFI) stage 2 (DFIS2) occurred more commonly in clinical practice in comparison with CEI, DFI mainly involved posterior soft tissue such as ligaments and parts of the intervertebral disc, whereas not bone [1]. Furthermore, compared to DFIS2, UCEI showed significantly higher rates of coronal malalignment and vertebral body destruction. This indicated that higher injury energy seemed to be associated with the latter one, resulting in more severe, unstable and poor clinical prognosis. Considering the main injury mechanism of compression-extension in Allen’s classification, it is also reasonable to observe the higher frequency of ALL and disc injuries. Currently, FE analysis is commonly known as an important method for biomechanical investigations. FE models could simulate different stages of clinical diseases for the needs of clinical practice limitation, and also to repeat treatment experiments theoretically to study impact responses in supplement of cadaveric tests. Thus, it was of potentially important clinical significance to simulate FE model of UCEI and investigate the effectiveness of surgical procedures.

In previous studies, posterior cervical fixation techniques have been developed, compared and validated in the long-term clinical relevant setting and biomechanical tests: (1) LS fixation technique: Heller et al. [33] suggested that the LS technique reconstructed the stability of the middle and posterior column of the lower cervical spine by penetrating the bilayer cortex with greater pullout strength than the monolayer cortex. What’s more, screw inserted by LS technique had high stress concentration at the actual caprod-screw interface, but less than 100 MPa of von Mises stress [34]. The LS fixation constrained motion in flexion as a tension band and limits motion in extension to some extend due to rigidity of rods, instead of constraining the motion in axial rotation like most pedicle screw fixations [35]. (2) PS fixation technique: Cervical pedicle is the strongest structure of cervical vertebra. Cortical bone around the pedicle is tubular, with a small amount of cancellous bone in the middle. The technique is based on the pedicle insertion screw with good three-column holding power. In addition, the ROM of the model after the reconstruction of three-column damage was significantly reduced, that is, the stability was significantly increased [34]. (3) TS fixation technique: TS technique has been used as an alternative technique to achieve posterior cervical spine stability in the lower cervical spine [30]. Additionally, it may be adequate for fixation in the lower cervical spine due to significantly higher pullout strength than lateral mass screws [36].

To the best of our knowledge, the current study is the first biomechanical study using FE model simulating a UCEI and analysis various internal fixations in the injured model. In this model with interbody bone graft fusion, the best stable construct was PS (bilateral) technique in flexion, which also enjoyed the best implant stress distribution. There was no significant difference of TS in comparison with LS and PS (unilateral) technique in the presence of ACDF. The LS and PS fixation techniques were widely used for SCS reduction and stabilization. From this experiment, the authors found that bilateral PS provided the best anti-compression strength and did not differ from other constructs. The bilateral PS proved the best anti-sheer ability and there was also no obvious difference between other constructs. In terms of implant stress, the bilateral PS exhibited advantage over the other groups.

The stability of the UCEI models following various fixations could be assessed by measuring the ROM, axial compression displacement and anterior shear displacement of C4/C5 local segments under all conditions (flexion, extension, lateral bending and axial rotation). Intersegmental motions analyses showed that the intersegmental motion of the C4/C5 segment significantly decreased following the rigid attachment of the internal fixation system (Fig. 5) compared with intact model. Despite some motion was still present, all constructs yielded good stability and there was no significant difference among different fixation techniques. In other words, the intrinsic strength of the TS fixation using single screw could provide immediate stabilization in comparison with other posterior fixations.

In general, the risk of implant breakage or screw loosing depends mainly on the amount of stresses in the implants, and on their alterations. More specifically, the higher stress level may dramatically decrease the use cycle of implants and low-cycle fatigue cracks are more likely to happen. Wang et al. [17] reported that the failure strength of titanium alloy (TC 4) was about 800-950 MPa, and the implant was likely to fail instantly under 600 MPa stress. The current study showed that screws inserted by TS technique had high stress concentration but much less than 600 MPa (251.47 MPa) at the middle part of the screw when performing combined TS and ACDF under different ROMs, which indicated that immediate stability was enabled to obtain and there was nearly no risk of screw breakage. In addition, under flexion-extension conditions, the results showed that the variability of von Mises stress in the ACDF device was obvious larger than combined fixation devices, and there were no significant differences under the other conditions. The maximum variability and stress-level of von Mises were analysed as a measurement a measurement of the potential for fracture due to different fixation techniques based on the assumption that maximum variability and high stress-level concentration results in greater possibility of fixation device fracture. According to the relevance of the maximum variability and stress-level of von Mises, our study suggested that the combination of posterior fixation and ACDF provided a circumstantial fixation and all constructs yielded good stability.

The authors further compared different kinds of screws for seeking a more minimally effective invasive internal fixation. Bilateral PS exhibited advantage over the other groups in terms of less ROM and stress distribution, which was in good agreement with previous studies. Interestingly, we did not observe such an obvious difference in our study for either LS, unilateral PS or TS. In other words, TS technique was stable enough and not worse than LS or unilateral PS. The major factor, we speculated, was the result of breaking the mobility of the zygapophysial joint by TS technique. Lee et al. [37] compared the acute stability of unilateral TS combined with an ACDF construct. Their study indicated that adjunctive unilateral TS fixation to an ACDF construct increased spinal stability significantly in the lower cervical spine. Other researchers also investigated that combined ACDF with TS, the posterior fixation, a “weak” fixation, constrained motion in flexion-extension effectively and was comparable with or replace the LS construct, a “strong” fixation, in all directions [36]. The result supported the idea that a “strong-weak” combination might be equivalent to “strong-strong” combination in posterior-anterior fixation of cervical spine.

In addition, PS fixation technique needs a high learning curve and is technically demanding in clinical practice. Also, it is generally considered a high surgical risk as the result of the potential to seriously injure the spinal cord, nerve roots, or vertebral arteries [38]. Whereas TS technique should be less technically demanding to acquire with less risk to the patient and can decrease intraoperative blood loss, paraspinal muscles injury, the postoperative neck pain and infection rate [16]. So, from technique complexity and neurologic risk point of view, the authors believed that TS technique followed by ACDF was superior to combined PS with ACDF for minimally invasive surgical treatment of UCEI.

It was reported that some surgeons preferred ACDF alone rather than combined cervical fixation technique in treating UCEI. The anterior procedure was well accepted for its less invasiveness and better ventral decompression and stabilization in the management of SCS injury. Nevertheless, Johnson et al. [39] described a 13% radiographic failure rate for anterior plate fixation in patients with flexion injuries of the SCS, including 75% with bilateral facet injuries. The authors postulated that the single ACDF was not sufficient for UCEI with the following reasons: (1) Literatures revealed that it was hard to take effective measures of reduction with skull traction for UFJF compared to bilateral facet joint fracture of SCS [2]. (2) Posterior procedure had advantage over anterior in relieving significant symptoms of nerve root compression often happened in the patients with UFJF of CEI. (3) From the biomechanical point of view, this current study indicated that it was not stable enough performed by ACDF alone. Specifically speaking, the results of a wider ROM (2.36° in flexion, around 1.00° in axial rotation) and anterior shear displacement (0.30 mm) and a higher maximum von Mises stress (nearly 500 MPa), which might lead to increased shear forces between fracture zone, possibly resulting in implant failure.

Based on the comparison of current five fixation techniques, the results suggested that, on the one hand, ACDF alone provided poor stability and not suitable in treating UCEI. The TS technique provided better stability and did not differ from single-sided LS and PS techniques. Although a high stress distribution might dramatically decrease the use cycle and low-cycle fatigue cracks were more likely occur in the long term, it was more stable than intact or injured model and this immediate stabilization was strong enough for sequential anterior decompression and fixation during operation. On the other hand, anterior plate fixation with interbody graft was insufficient and circumstantial fixation was recommended. What’s more, in comparison with PS, it was a safe and convenient way to perform TS fixation technique. And compared to LS, TS had the advantage in preserving the activity of adjacent segments and having significantly higher pullout strength [36]. Thus, the combined procedure of posterior TS fixation followed by additional ACDF could yield great stability.

The present study has several limitations. Firstly, it is important to note that the loading conditions without taking muscle force into account were highly idealized, and the models were simulated UCEI immediately postoperatively while neglecting bone fusion or postoperative orthosis which could help restrict the motion of involved segments of the spine. Secondly, we assumed the material properties of bone and implant were elastic and set no failure point. Thirdly, degeneration of cervical spine was not considered, and the anatomy and the material properties were modelled based on the healthy condition. Despite this, we believe a comparison between different fixation techniques is still possible and analysis results have clinic instructive significance to cervical fixation technique.

## Conclusions

Based on our biomechanical findings, in the case of UCEI, ACDF-only procedure has proven poor stability, high structural stress and risk of nail breakage. Combined TS and ACDF technique would be a reasonable treatment option to acquire an ideal immediate stabilization. However, clinical aspects must also be regarded when choosing a fixation method for a specific patient.

## Data Availability

Summarized data have been presented in this manuscript. The raw data for this study are not publicly available due to a planned secondary analysis but are available from the corresponding author on reasonable request.

## Abbreviations

SCS: Subaxial cervical spine
CEI: Compression-extension injury
UCEI: Unstable compression-extension injury
UFJF: Unilateral facet joint fracture
PS: Pedicle screw
LS: Lateral mass screw
TS: Transarticular screw
ACDF: Anterior cervical discectomy and fusion
FE: Finite element
ALL: Anterior longitudinal ligament
PLL: Posterior longitudinal ligament
CL: Capsular ligament
LF: Ligamenta flava
ISL: Interspinous ligaments
ROM: Range of motion
DFI: Distractive flexion injuries
DFIS2: Distractive flexion injuries stage 2
